# Nitric Oxide Regulates Neuronal Activity via Calcium-Activated Potassium Channels

**DOI:** 10.1371/journal.pone.0078727

**Published:** 2013-11-13

**Authors:** Lei Ray Zhong, Stephen Estes, Liana Artinian, Vincent Rehder

**Affiliations:** Biology Department, Georgia State University, Atlanta, Georgia, United States of America; University of Waterloo, Canada

## Abstract

Nitric oxide (NO) is an unconventional membrane-permeable messenger molecule that has been shown to play various roles in the nervous system. How NO modulates ion channels to affect neuronal functions is not well understood. In gastropods, NO has been implicated in regulating the feeding motor program. The buccal motoneuron, B19, of the freshwater pond snail *Helisoma trivolvis* is active during the hyper-retraction phase of the feeding motor program and is located in the vicinity of NO-producing neurons in the buccal ganglion. Here, we asked whether B19 neurons might serve as direct targets of NO signaling. Previous work established NO as a key regulator of growth cone motility and neuronal excitability in another buccal neuron involved in feeding, the B5 neuron. This raised the question whether NO might modulate the electrical activity and neuronal excitability of B19 neurons as well, and if so whether NO acted on the same or a different set of ion channels in both neurons. To study specific responses of NO on B19 neurons and to eliminate indirect effects contributed by other cells, the majority of experiments were performed on single cultured B19 neurons. Addition of NO donors caused a prolonged depolarization of the membrane potential and an increase in neuronal excitability. The effects of NO could mainly be attributed to the inhibition of two types of calcium-activated potassium channels, apamin-sensitive and iberiotoxin-sensitive potassium channels. NO was found to also cause a depolarization in B19 neurons *in situ*, but only after NO synthase activity in buccal ganglia had been blocked. The results suggest that NO acts as a critical modulator of neuronal excitability in B19 neurons, and that calcium-activated potassium channels may serve as a common target of NO in neurons.

## Introduction

Nitric oxide (NO) serves as an unconventional membrane-permeable messenger molecule in the nervous systems of vertebrates and invertebrates, where it has been implicated in various cellular processes, including neuronal migration [1], synaptogenesis [2], long-term potentiation [3], and memory formation [4], [5]. One mode of action by which NO has been shown to elicit its effects in neurons is by modulating ionic conductances [6]. Among ion channels, calcium (Ca^2+^) channels [7], potassium (K^+^) channels [8], and HCN channels [9] have been shown to be targets of NO signaling. How NO modulates membrane channels to affect aspects of the functional output of neuronal circuits is of central interest in many systems.

Progress has been made towards understanding the role of NO signaling in gastropods [10]. Using isolated neurons from the buccal ganglion of *Helisoma trivolvis*, NO has been characterized as a regulator of neurite outgrowth and growth cone motility [11], [12]. Application of NO-donors to the buccal neuron B5 slowed the advance of growing neurites [13], whereas growth cone filopodia underwent transient elongation [11], suggesting a role for NO in neuronal pathfinding during development and regeneration. NO has also been shown to modulate neuronal excitability in B5 neurons by selectively affecting ion channels, such as K^+^ and Ca^2+^ channels [14], [15]. On the level of neuronal circuitry and animal behavior, NO has been shown to be important in aerial respiration and long-term associative memory in *Lymnaea* [4], [16], [17] and in feeding behaviors in *Aplysia* [18]–[21] and *Lymnaea* [22]–[24].

Gastropod feeding is driven by central pattern generators [25], [26], and NO has been implicated in regulating the feeding motor program [22]–[24]. Buccal neuron B19 in *Helisoma* is a bilaterally symmetric motor neuron that innervates muscle groups in the radula [26], [27]. The somata of B19 neurons are located in the vicinity of NO-producing neurons [12], [28], suggesting that NO might affect B19 neurons by volume transmission. The goal of the current study was to investigate potential modulatory effects of NO on the electrical activity of B19 neurons, to identify the ion channels affected by NO, and to determine if NO acted on the same or a different set of ion channels than in the previously characterized buccal neuron (B5) involved in snail feeding. To eliminate possible indirect effects contributed by other cells, and to allow cell type specific responses to NO to be investigated in isolation, we performed most experiments at the single cell level in cultured B19 neurons, where the source of NO is well controlled and the potential intracellular targets affected by NO can be investigated directly. We then compared the effects of NO with those on B19 neurons located in ganglia.

We found that NO donors caused a prolonged depolarization of the membrane potential and an increase in neuronal excitability in cultured B19 neurons. This effect of NO could be attributed in large part to the inhibition of Ca^2+^-activated K^+^ channels, with apamin-sensitive K (SK) channels serving as the main target, and their inhibition by NO fully accounting for the sustained depolarization. Inhibition by NO of iberiotoxin (IbTX)-sensitive K (BK) channels contributed an early and transient effect to the overall depolarization. Moreover, NO elicited a similar depolarizing effect on B19 neurons in intact ganglia, but only after ganglionic NO synthase (NOS) activity had been inhibited pharmacologically. Our data support the notion that NO can serve as a key modulator of neuronal activity, and that Ca^2+^-activated K^+^ channels may be a common target of NO signaling via volume transmission.

## Materials and Methods

### Animals

Freshwater pond snails (*Helisoma trivolvis*) were kept in aerated aquaria (10 gallons) containing filtered water at room temperature on a 12 h light-dark cycle. Vegetable-based algae wafers (Hikari, Doctors Forster and Smith) and organic lettuce were used to feed snails once every day. Middle-sized animals with a shell diameter of 15–20 mm were chosen for the experiments.

### Neuronal culture

Identified B19 neurons were isolated from the buccal ganglion of *Helisoma* and plated into Falcon Petri dishes as previously described [29]. Briefly, neurons were plated onto poly-L-Lysine (hydrobromide, MW, 70–150 kDa, 0.25 mg/ml; Sigma, St. Louis, MO, USA)-coated glass coverslips attached to the bottom of 35-mm cell culture dishes (Falcon 1008). B19 neurons were kept in conditioned medium at room temperature. Conditioned medium was prepared by incubating two *Helisoma trivolvis* brains per 1 mL of Leibowitz L-15 medium (Invitrogen, Carlsbad, CA, USA) for 4 days. B19 neurons were used for experiments 24–48 hours after plating. The composition of L-15 medium was as follows (mM): 44.6 NaCl, 1.7 KCl, 1.5 MgCl_2_, 0.3 MgSO_4_, 0.14 KH_2_PO_4_, 0.4 Na_2_HPO_4_, 1.6 Na pyruvate, 4.1 CaCl_2_, 5 HEPES, 50 µg/ml gentamicin, and 0.15 mg/ml glutamate in distilled water, pH 7.4.

### Electrophysiology

Recordings from cultured *Helisoma* B19 neurons in whole-cell current-clamp mode were obtained as described previously [30]. The patch electrodes were pulled from borosilicate glass tubes (OD 1.5 mm; ID 0.86 mm; Sutter instruments) on a Sutter instruments micropipette puller (P-87) and heat polished (Micro Forge MF-830; Narishige) with a resistance of about 3–8 MΩ. Neurons were recorded using 700B amplifiers (Molecular Devices, Union City, CA) and an analog-to-digital converter (Digidata 1440). Data acquisition and analysis were performed using pClamp software version 10 (Molecular Devices). Recorded signals were digitized at 10 kHz, and filtered at 1 kHz. Current-clamp configuration was used to record membrane potential, firing properties, and evoked action potentials (APs). Leibowitz L-15 medium was normally used as extracellular recording solution. In some experiments, L-15 medium was replaced with normal saline containing in (mM): 51.3 NaCl, 1.7 CaCl_2_, 1.5 MgCl_2_, and 5 HEPES, pH 7.3–7.4 (127 mOsm). Intracellular recording solution contained (mM): 54.4 K-aspartate, 2 MgCl_2_, 5 HEPES, 5 Dextrose, 5 ATP, 0.1 EGTA (127 mOsm). The concentration of EGTA (100 µM) had been determined empirically in a previous publication (Artinian et al. 2010) as a buffer concentration that did not affect the spontaneous firing activity in B5 neurons in whole cell patch clamp recordings. The membrane potential was not corrected for liquid junction potential, which we calculated to be approximately 13 mV. Mixed tetraethylammonium (TEA) chloride and 4-aminopyridine (4AP) solution was made by replacing 20 mM NaCl with 20 mM TEA(Cl) and adding 5 mM 4AP right before the experiment. Solution replacement was achieved through a gravity-based perfusion system (Warner Instruments). The resting membrane potential of a spontaneously firing neuron was determined by measuring the value at the plateau of the depolarization phase before the membrane potential reached threshold. Measurements of the effects of NO donors, NOC7 or DEA/NO, on membrane potential were made at two separate time points in order to account for what appeared to be an initial, slightly larger increase, followed by a sustained depolarization. The initial phase was measured at approximately 30 s after NOC7 stimulation, whereas the plateau phase was measured at 3 min after NOC7 application. Neuronal excitability was tested by injecting depolarizing current with amplitudes of +20 and +100 pA for 1 s. Analysis of the properties of evoked APs was achieved by using the ‘threshold search’ function of Clampfit (pClamp 10, Molecular Devices).

Recordings of baseline currents and voltage-gated Ca^2+^ currents in response to stimulation with NO donors were achieved in the whole-cell voltage-clamp configuration, as described previously [31]. As for the study of NO-induced baseline currents, the membrane potential was held at −50 mV. To study the input resistance (R_in_), membrane voltage was stepped to −60 mV for 0.5 s (ΔV = 10 mV), and the resulting current was used to calculate R_in_. Signals were smoothed using a 31-point boxcar filter. To characterize total Ca^2+^ currents, the membrane potential was held at −60 mV and stepped from −60 mV to + 60 mV for 500 ms and 10 mV increments. Extracellular solution contained (in mM): 10 CaCl_2_, 45.7 TEA(Cl), 1 MgCl_2_, 2 4AP, 10 HEPES, pH 7.4 (TEA-OH); internal solution (in mM): 29 CsCl, 2.3 CaCl_2_, 2 MgATP, 0.1 GTP-Tris, 11 EGTA, 10 HEPES. The internal solution was adjusted to pH 7.4 with CsOH. Recordings were filtered at 5 kHz (−3 dB, 4 pole Bessel filters). Currents were analyzed by normalizing the peak inward current for each cell to the cell capacitance (pA/pF).

Recordings from B19 neurons located within buccal ganglia were achieved in whole-cell current-clamp mode. Ganglia were pinned down in a dissection chamber containing L-15 medium. The ganglionic sheath in the vicinity of the B19 neuron was cut open to expose the neuron using a fine microknife. Neurons in ganglia were patch-clamped and recorded in a similar way as described for cultured neurons earlier. Inhibition of NOS activity in ganglia was achieved by incubating whole ganglia in a solution containing two NOS inhibitors, 1 mM L-NAME and 100 µM 7-NI, for 1 to 2 hours, before establishing the patch clamp configuration.

### Pharmacological agents

The NO donor, 3-(2-hydroxy-1-methyl-2-nitrosohydazino)-N-methyl-1-propyanamine (NOC7, Calbiochem) was dissolved in 100 mM NaOH to make a 100 mM stock solution. Diethylamine NONOate (DEA/NO, Calbiochem), cadmium chloride (CdCl_2_, Sigma), iberiotoxin (IbTX, Sigma), apamin (Alomone labs), and NG-nitro-L-arginine methyl ester (L-NAME, Calbiochem), were dissolved in distilled H_2_O to make 100 mM, 1 M, 200 µM, 1 mM, 1 M stock solutions, respectively. 7-Nitroindazole (7NI, Calbiochem) was dissolved in dimethylsulfoxide (DMSO, Sigma) to make 100 mM stock solution. For patch clamp experiments, stock solutions were mixed with 200 µl extracellular solution removed from the recording dish, gently added back around the periphery of the dish, and aspirated for 5 times using a 200 µl pipette to equilibrate the drugs to their final concentrations. The K^+^ channel blockers, tetraethylammonium (TEA, Sigma) and 4-aminopyridine (4AP, Sigma), were prepared directly in the extracellular solution. The choice of pharmacological blockers was based on their successful prior usage in *Helisoma* [14], [30].

### Statistical analysis

All data were expressed as mean ± SEM. Comparisons between two individual groups were made with either the Mann-Whitney U-test or the two-sample t-test, and comparisons between two paired groups were achieved by the paired-sample Wilcoxon signed-rank test using Origin Data Analysis and Graphing software (OriginLab Corporation, Northampton, MA). Significant differences are indicated as *P<0.05, **P<0.01, and ***P<0.001.

## Results

### Nitric oxide depolarizes the membrane potential of B19 neurons

**Figure 1:**
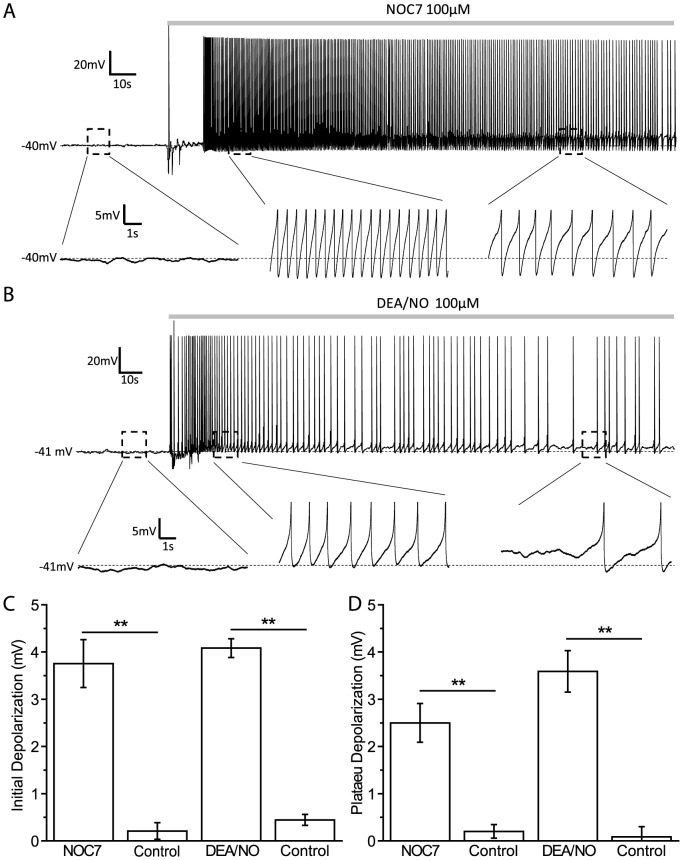
NO causes membrane potential depolarization in B19 neurons. A: A silent B19 neuron was depolarized and started firing after treatment with NOC7 (100 µM, bath application; gray bar). Note electrical recording artifact upon drug addition. Enlarged areas of interest below the main recording trace (marked by black dashed boxed) show details of the recording (note that APs are clipped to emphasize membrane depolarization). The initial depolarization to NOC7 at 30 s was stronger than that during the plateau phase at 3 min. B: Representative data showing membrane potential depolarization of B19 neurons by another NO donor, DEA/NO (100 µM). C: Quantification of the changes in membrane potential during the initial phase such as shown in A and B. Both NOC7 and DEA/NO caused a significant depolarization compared to that of their vehicle control groups. D. Quantification of the plateau depolarization showing that both NOC7 and DEA/NO caused significant depolarization during the plateau phase.

Isolated B19 neurons from the buccal ganglion of *Helisoma trivolvis* were used for whole-cell patch-clamp experiments 24–48 hour after plating, at which time all neurons had well-developed neurites with growth cones at their tips. 74.6 percent of B19 neurons recorded (44 out of 59) were silent with a resting membrane potential at −41.2±0.7 mV, whereas the rest of B19 neurons (25.4%, 15 out of 59) fired spontaneous action potentials (APs) and had a slightly more depolarized membrane potential of −38.3±0.7 mV (P<0.05; Two-sample t-test). We first asked how nitric oxide (NO) might affect the electrical activity of B19 neurons. The NO donor, NOC7 (100 µM; half life: 10 min at 22°C, pH 7.4, Calbiochem), was used to activate NO signaling [13], [14], [32]. Despite their initial differences in membrane potential, all B19 neurons responded to NOC7 with depolarization. In spiking neurons, as well as in neurons in which the depolarization was large enough to bring neurons to the spiking threshold, neurons responded with a phasic increase in firing frequency, followed by sustained firing [Fig. 1(A)]. To include all neurons in the analysis, we decided to quantify the amount of depolarization resulting from the stimulation with NOC7. To account for what appeared to be an initial, slightly larger increase, followed by a sustained depolarization, we measured these effects of NO on membrane potential separately. The initial phase, measured approximately 30 s after NOC7 stimulation, showed a slightly stronger depolarization compared to the plateau phase, measured at 3 min after NOC7 application (initial phase: +3.8±0.5 mV, n = 8; plateau phase: +2.5±0.4 mV, n = 8, P<0.01; Paired-sample Wilcoxon signed-rank test) [Fig. 1(A, C and D)]. Both phases of the effect of NOC7 were significantly different from the solvent control, which had no effect (initial phase: +0.2±0.2 mV, n = 6, P<0.01, compared to NOC7; plateau phase: +0.2±0.1 mV, n = 6, P<0.01, compared to NOC7; Mann-Whitney U-test) [Fig. 1(C and D)].

We next wanted to independently confirm the effect of NO on membrane potential by using another NO donor, DEA/NO, which has been used successfully on B5 neurons in our system [15]. DEA/NO releases NO with a half-life of 16 min at 22°C and pH 7.4 (Calbiochem). Similar to the effects seen with NOC7, bath application of 100 µM DEA/NO caused an initial depolarization (+4.1±0.2 mV, n = 6) and a plateau response (+3.6±0.4 mV, n = 6) [Fig. 1(B to D)]. Both phases of the DEA/NO effect were significant compared to the solvent control (initial phase: +0.4±0.1 mV, n = 6, P<0.01, compared to DEA/NO; plateau phase: +0.1±0.2 mV, n = 6, P<0.01, compared to DEA/NO; Mann-Whitney U-test) [Fig. 1(C and D)].

Taken together, stimulation with NO by the application of NOC7 or DEA/NO caused a depolarization of the membrane potential in B19 neurons, with a relatively stronger initial phase and a sustained plateau phase. We next wanted to determine the source of the depolarization in response to NO and first considered the opening of Ca^2+^ channels as a likely cause.

### The NO donor NOC7 does not affect voltage-gated Ca^2+^ channels (VGCCs)

**Figure 2:**
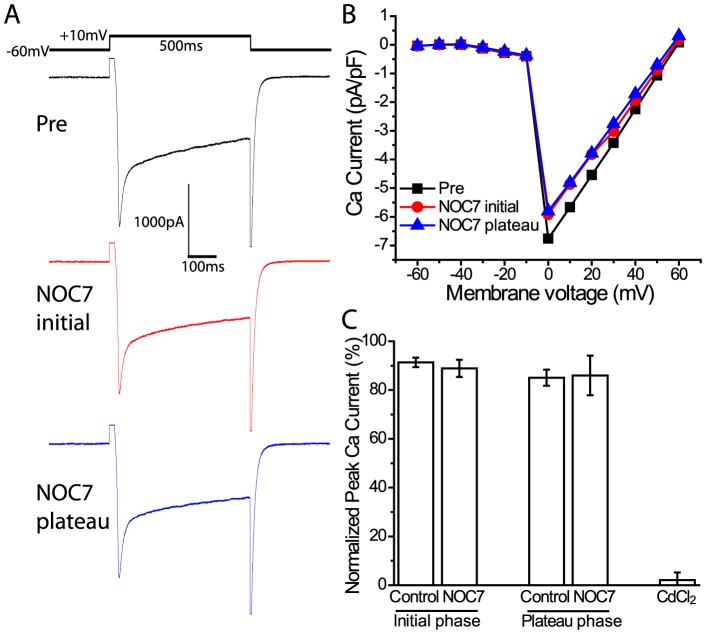
Voltage-gated Ca^2+^ channels are not affected by NOC7. Ca^2+^ currents were recorded in whole-cell voltage-clamp mode. Voltage steps from a holding potential of −60 mV to +60 mV were applied in 10 mV increments. A: Representative traces of Ca^2+^ currents evoked by a voltage step from −60 mV to +10 mV before (upper), during the initial phase (middle), and during the plateau phase of treatment with NOC7 (100 µM, lower). B: Representative I–V plot of Ca^2+^ current measured at the peak amplitude and expressed as normalized Ca^2+^ current (pA/pF) before and after NOC7 application. Note that NOC7 did not have an obvious effect on Ca^2+^ currents. C: Quantification of the effect of NO on Ca^2+^ currents showing that treatment with NOC7 did not have a significant effect on normalized peak currents compared to control groups during both initial and plateau phases. Subsequent application of the Ca^2+^ channel blocker CdCl_2_ (100 µM) fully eliminated Ca^2+^ currents.

To test possible effects of NOC7 on VGCCs, we recorded Ca^2+^ currents in B19 neurons directly in the whole-cell voltage-clamp configuration, as previously described [15]. We used a Na-free extracellular medium combined with a K-free intracellular solution to isolate Ca^2+^ currents and applied voltage steps from −60 to +60 mV for 500 ms and 10 mV increments to evoke Ca^2+^ currents. The maximal Ca^2+^ current was not affected by treatment with 100 µM NOC7 [Fig. 2(A and B)]. The normalized peak Ca^2+^ currents during both the initial and the plateau phases of depolarization induced by NOC7 were not significantly different from the solvent control (initial phase: NOC7: 88.9±3.6%, n = 5 vs control: 91.4±1.9%, n = 4, P = 0.71; plateau phase: NOC7: 86.0±8.1%, n = 5 vs control: 85.1±3.3%, n = 4, P = 1; Mann-Whitney U-test) [Fig. 2(C)]. Subsequent treatment with 100 µM CdCl_2_, a prominent inhibitor of VGCCs, fully blocked the current (2.1±3.1%, n = 5) [Fig. 2(C)], suggesting that the current recorded was indeed a Ca^2+^ current. Therefore, an opening of VGCCs in response to extrinsic NO stimulation could be ruled out as the source of depolarization.

### NOC7 induces an inward current and increases R_in_ in B19 neurons

**Figure 3:**
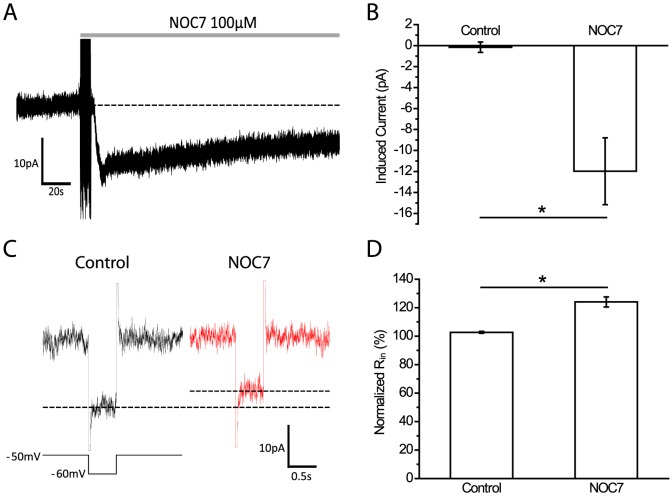
NO induces an inward current and increases input resistance. A: Representative recording of a B19 neuron showing that treatment with NOC7 (100 µM) immediately elicited an inward current (holding potential at −50 mV). B: Quantification of the maximal NO-induced current showing that NOC7 evoked a significant inward current compared to the control group. C: Comparison of currents evoked by a voltage step of −10 mV for 0.5 s before and after treatment with NOC7 (100 µM). Note the reduction in the current after NOC7 application (indicated by dashed line). D: Quantification of normalized R_in_ for vehicle control and NOC7 groups. R_in_ was normalized to pretreatment values and is expressed in percent. NOC7 significantly increased the R_in_ in B19 neurons.

We next tested the effect of NO on membrane currents under voltage-clamp conditions. Treatment with 100 µM NOC7 immediately induced an inward current [Fig. 3(A)]. The peak amplitude of the NOC7-induced current was −12.0±3.2 pA (n = 5), which was significantly larger than the control group (−0.2±0.5 pA, n = 5, P<0.05, compared to NOC7; Mann-Whitney U-test) [Fig. 3(B)]. Next, we determined the input resistance (R_in_) before and after application of 100 µM NOC7 and found that the current evoked by a voltage step of −10 mV was reduced after treatment with NOC7 [Fig. 3(C)]. The analysis of R_in_ showed that NOC7 significantly increased the R_in_ of B19 neurons (NOC7: 124.0±3.5%, n = 5 vs control: 102.7±0.7%, n = 5, P<0.05; Mann-Whitney U-test) [Fig. 3(D)], suggesting the closure of membrane channels in response to NO. Considering that NOC7 caused an inward current and an increase in R_in_, we next considered that K^+^ channels may serve as main targets of NO.

### Inhibition of K^+^ channels fully blocks the depolarizing effects of NOC7

**Figure 4:**
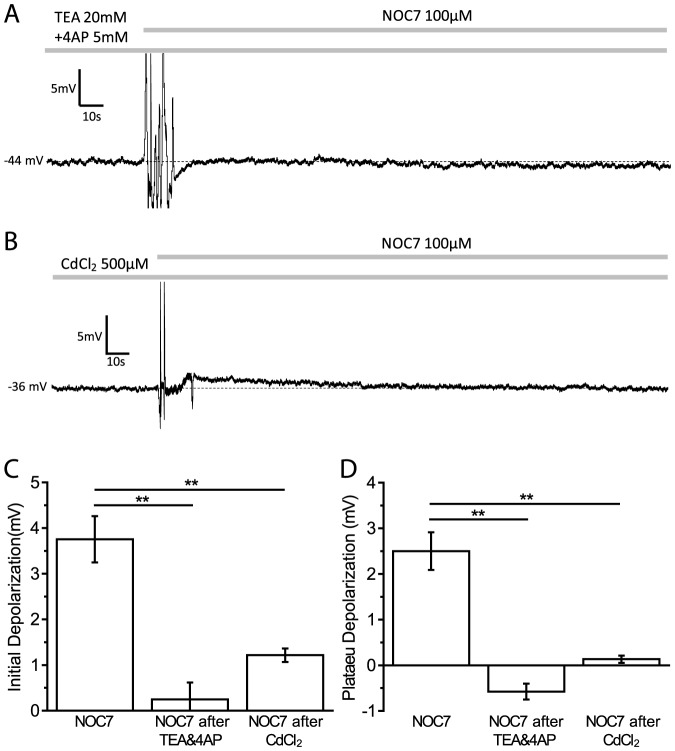
Ca^2+^-activated K^+^ channels mediate NO-induced depolarization. A: Representative recording of a B19 neuron pretreated with a cocktail of the K^+^ channel blockers TEA (20 mM) and 4AP (5 mM), and subsequently treated with NOC7 (100 µM). Inhibition of K^+^ channels completely blocked the depolarizing effect of NOC7. B: Example of a B19 neuron pretreated with CdCl_2_ (500 µM) before and after treatment with NOC7 (100 µM). CdCl_2_ was used to block Ca^2+^ influx, and indirectly inhibited the activation of Ca^2+^-activated K^+^ channels. Note that NOC7 had only a small depolarizing effect on membrane potential during the initial phase, whereas any depolarization during the plateau phase was fully inhibited in the presence of CdCl_2_. C: Quantification of the initial depolarization showing that pretreatment with TEA (20 mM) and 4AP (5 mM) fully blocked the depolarizing effect of NOC7, whereas CdCl_2_ (500 µM) significantly inhibited the effect of NOC7 during the initial phase. D: Pretreatment with TEA and 4AP and with CdCl_2_ prevented the NOC7-induced depolarization during the plateau phase.

In order to investigate the contribution of K^+^ channels in the NO-induced depolarization, we first used a cocktail of 20 mM TEA and 5 mM 4AP to block the majority of K^+^ channels. While this treatment depolarized the membrane potential instantly as expected (+4.7±1.6 mV, n = 4) [data not shown], it also completely blocked any additional effect of a subsequent treatment with NOC7 (100 µM) (initial phase: NOC7 after TEA&4AP: +0.2±0.4 mV, n = 5, P<0.01, compared to NOC7; plateau phase: NOC7 after TEA&4AP: −0.6±0.2 mV, n = 5, P<0.01, compared to NOC7; Mann-Whitney U-test) [Fig. 4(A, C and D)]. Interestingly, the degree of depolarization obtained by inhibition of a majority of K^+^ channels (plateau phase: TEA&4AP: +4.7±1.6 mV, n = 4) was not significantly different from that seen after treatment with NOC7 (P = 0.27; Mann-Whitney U-test), suggesting that the effect of NO on membrane potential could likely be explained by an inhibitory effect of NOC7 on K^+^ channels. We next wanted to determine the class of K^+^ channels that mediated the NOC7-induced depolarization.

### NO depolarizes the membrane potential in a Ca^2+^-dependent manner

To test for the involvement of Ca^2+^-activated K^+^ channels in the depolarizing response to NOC7, we first used CdCl_2_ to block VGCCs and then applied NOC7. 500 µM CdCl_2_ resulted in a depolarization of the membrane potential (CdCl_2_: +2.9±0.3 mV, n = 4 vs control: +0.1±0.2 mV, n = 6, P<0.05; Mann-Whitney U-test) [data not shown], suggesting a contribution of Ca^2+^ influx to the resting membrane potential, likely mediated via Ca^2+^-activated K^+^ channels. Interestingly, the plateau effect of CdCl_2_ was not significantly different from that seen after treatment with NOC7 (P = 0.44; Mann-Whitney U-test), suggesting that the effect of NO on membrane potential might be largely explained by an inhibitory effect of NOC7 on Ca^2+^-activated K^+^ channels. Indeed, subsequent application of 100 µM NOC7 caused only a small depolarization during the initial phase (NOC7 after CdCl_2_: +1.2±0.1 mV, n = 4, P<0.01, compared to NOC7; Mann-Whitney U-test) [Fig. 4(B and C)], whereas the plateau phase of the NO effect was completely blocked in the presence of 500 µM CdCl_2_ (NOC7 after CdCl_2_: +0.1±0.1 mV, n = 4, P<0.01, compared to NOC7; Mann-Whitney U-test) [Fig. 4(B and D)].

Taken together, the results suggested a main role for Ca^2+^-activated K^+^ channels as cellular targets of NO stimulation. We next investigated which classes of Ca^2+^-activated K^+^ channels might be targeted by NOC7 using pharmacological tools.

### IbTX-sensitive K^+^ channels partially contribute to the initial phase of the NO effect

**Figure 5:**
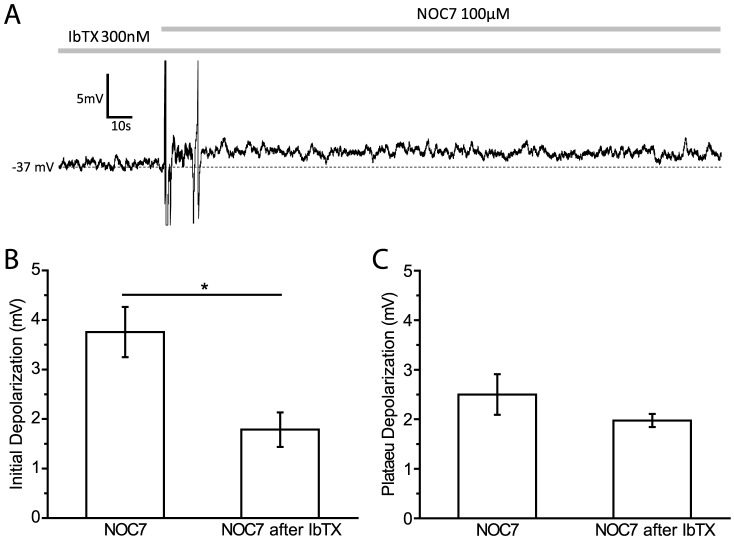
IbTX-sensitive BK channels partially contribute to the initial depolarization induced by NOC7. A: Representative recording of a B19 neuron pretreated with IbTX (300 nM) and after addition of NOC7 (100 µM). Note that NOC7 after IbTX caused a sustained depolarization with similar initial and plateau amplitudes. B: Quantification of the initial depolarization showing that the amplitude of membrane depolarization was significantly reduced in the NOC7 after IbTX group compared to NOC7 by itself. C: Quantification of the plateau depolarization in response to treatment shown in A. IbTX pretreatment did not affect the depolarizing effect of NO during the plateau phase.

Two subtypes of Ca^2+^-activated K^+^ channels have been reported in *Helisoma* to date: a large conductance (BK) channel and a small conductance (SK) channel, each with a distinct pharmacological profile and contribution to neuronal activity [14]. We first investigated the potential effect of NO on BK channels. BK channels can be inhibited pharmacologically by iberiotoxin (IbTX), a scorpion toxin that acts on the outer face of the channel [33]. IbTX has been used successfully in blocking BK channels in *Helisoma* B5 neurons [14]. 300 nM IbTX caused a slow depolarization, which reached a plateau at around 10 min (IbTX: +2.7±0.7 mV, n = 4 vs control: +0.1±0.2 mV, n = 6, P<0.05; Mann-Whitney U-test) [data not shown], suggesting that BK channels in B19 neurons are partially open at rest and help maintain the membrane potential at a hyperpolarized level. Subsequent application of 100 µM NOC7 in the presence of IbTX still caused additional depolarization, which was maintained throughout the recording, suggesting that NOC7 was acting on yet another conductance, in addition to BK channels [Fig. 5(A)]. During the early phase, in the presence of IbTX, NOC7 treatment was able to add an additional depolarization that was significantly smaller than the one produced by NOC7 itself (NOC7 after IbTX: +1.8±0.3 mV, n = 4, P<0.05, compared to NOC7; Mann-Whitney U-test) [Fig. 5(A and B)], suggesting that the initial depolarization by NO was mediated by at least two channels: an IbTX-sensitive K^+^ channel and a yet unknown channel. During the plateau phase, the depolarization in response to NOC7 in IbTX-pretreated neurons was not significantly different from NOC7 on its own (NOC7 after IbTX: +2.0±0.1 mV, n = 4, P = 0.73; Mann-Whitney U-test) [Fig. 5(A and C)], suggesting IbTX-sensitive BK channels did not significantly contribute to the sustained plateau effect of NO.

### Apamin-sensitive K^+^ channels are the main target of NO in depolarizing the membrane potential

**Figure 6:**
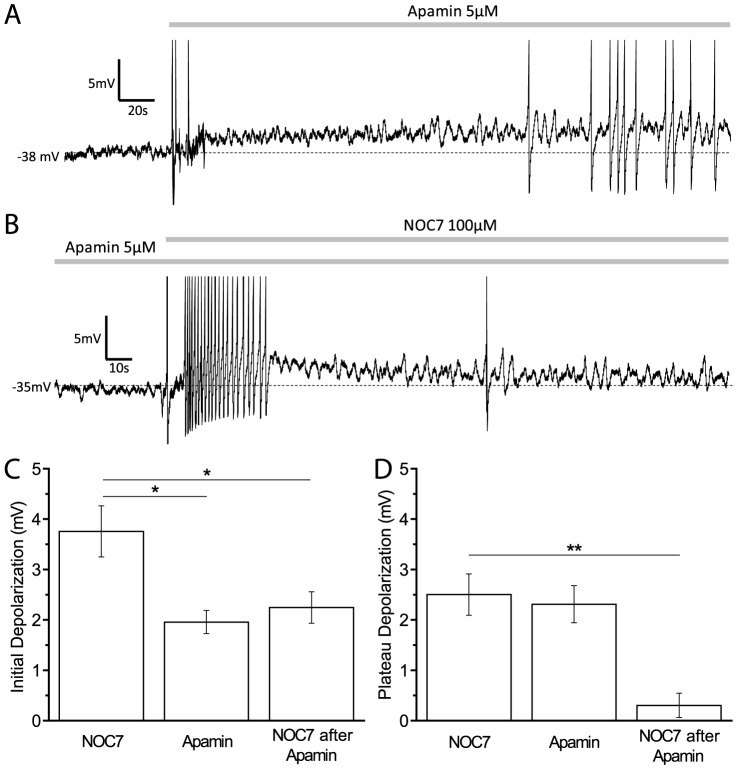
Apamin-sensitive SK channels are responsible for the main effect of NO on membrane potential. A: Representative recording of a B19 neuron before and after treatment with apamin (5 µM). Note that apamin application led to a sustained depolarization. B: Pre-incubation with apamin (5 µM) fully blocked the plateau depolarization normally seen by treatment with NOC7 (100 µM), but a small initial depolarization was still observed. C: Quantification of the initial depolarization such as shown in A and B. Apamin caused a depolarization, but the amplitude was significantly smaller than that of NOC7 group. NOC7 after pretreatment with apamin induced a significantly smaller depolarization than NOC7 by itself. D: Quantification of the plateau depolarization showing that treatment with NOC7 or apamin resulted in a similar depolarization. Subsequent application of NOC7 in the presence of apamin did not cause any additional depolarization during the plateau phase.

SK channels are known to be the target of NO in *Helisoma* B5 neurons [14], which raised the possibility that SK channels might be affected by NO in B19 neurons as well. Treatment with 5 µM apamin, a specific blocker of SK channels, instantly led to a sustained depolarization in B19 neurons [Fig. 6(A)]. Whereas the initial depolarization by apamin was slightly smaller than that by NOC7 treatment (Apamin: +2.0±0.2 mV, n = 5, P<0.05, compared to NOC7; Mann-Whitney U-test) [Fig. 6(C)], the sustained depolarization achieved by apamin was similar to that of NOC7 group (Apamin: +2.3±0.4 mV, n = 5, P = 0.71, compared to NOC7; Mann-Whitney U-test) [Fig. 6(A and D)]. The subsequent addition of NOC7 (100 µM) to apamin-treated neurons resulted in an additional, albeit transient depolarization (initial phase: NOC7 after apamin: +2.2±0.3 mV, n = 5, P<0.05; compared to NOC7; Mann-Whitney U-test) [Fig. 6(B and C)], suggesting that the initial depolarization partially resulted from another channel in addition to SK channels, probably IbTX-sensitive BK channels as demonstrated earlier. Interestingly, no additional depolarization could be achieved by NOC7 in the presence of apamin during the plateau phase (NOC7 after apamin: +0.3±0.2 mV, n = 5, P<0.01; compared to NOC7; Mann-Whitney U-test) [Fig. 6(B and D)]. These results suggested a main role for SK channels in the response to NO, with the sustained plateau effect elicited by NOC7 fully prevented by apamin.

### NO increases neuronal excitability

**Figure 7:**
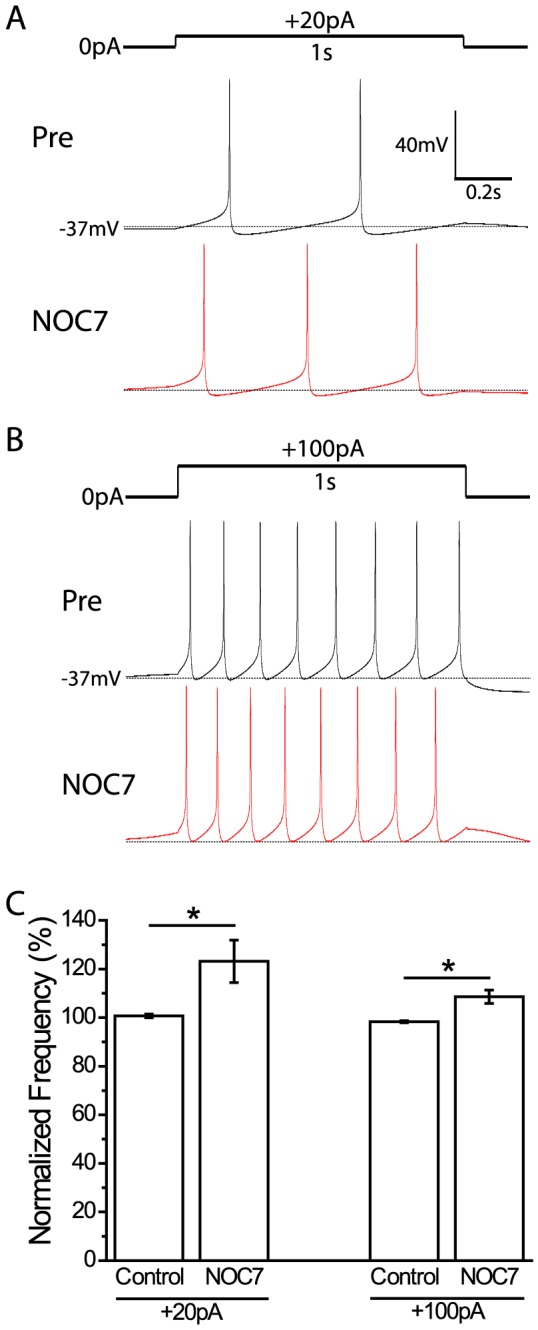
NOC7 increases the excitability of B19 neurons. A: Comparison of action potentials evoked by injecting depolarizing current (+20 pA, 1 s) before and after treatment with NOC7 (100 µM). Note that one more AP was induced after NOC7 application. B: Evoked APs in response to +100 pA current injection for 1 s before and after treatment with NOC7 (100 µM). Note that NOC7 application resulted in shortened inter-spike intervals. C: Quantification of normalized spike frequency for vehicle controls and NOC7 groups. The frequency of evoked APs after treatment was normalized to that before treatment. In both +20 pA and +100 pA current injection conditions, NOC7 caused a significant increase in the frequency of evoked APs.

Given its depolarizing effect on membrane potential, we next wanted to further investigate the effect of NO on neuronal excitability. Injections of depolarizing current steps for 1 s evoked APs in B19 neurons in a dose-dependent manner. In the example shown in Fig. 7(A), 100 µM NOC7 shortened the inter-spike interval, which allowed one more evoked AP to occur over the period of +20 pA current injection. Statistical analysis of the firing frequency of the evoked APs showed that B19 neurons significantly increased their firing frequency elicited by +20 pA current injections compared to the vehicle control (NOC7: 123.2±8.7%, n = 4 vs control: 100.7±0.8%, n = 4, P<0.05; Mann-Whitney U-test) [Fig. 7(C)]. The increased firing frequency induced by NO was maintained when a larger depolarizing current was applied (+100 pA: NOC7: 108.6±2.7%, n = 4 vs control: 98.3±0.4%, n = 4, P<0.05; Mann-Whitney U-test) [Fig. 7(B and C)]. Therefore, NO not only caused a depolarization of the membrane potential and increased firing frequency, but also led to a general increase in neuronal excitability of B19 neurons.

### NOC7 also depolarizes B19 neurons *in situ*, but only after inhibition of NOS in the ganglion

**Figure 8:**
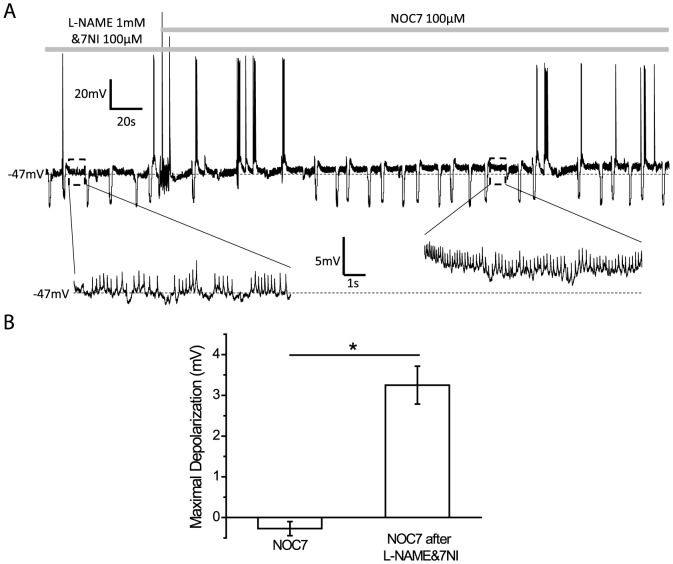
NO causes a depolarization in B19 neurons *in situ* in the presence of NOS inhibitors. A: A representative recording of a B19 neuron located within the buccal ganglion showing that treatment with NOC7 (100 µM) depolarized the membrane potential after the ganglion had been incubated in a solution containing two NOS inhibitors, L-NAME (1 mM) and 7NI (100 µM). Note that the membrane potential is enlarged at higher temporal resolution (highlighted by dashed black boxes) before and after the application of NOC7 to show the depolarization induced by NOC7. B: Quantification of maximal changes in the membrane potential. While NOC7, by itself, did not have an effect on the membrane potential of B19 neurons in intact ganglia, NOC7 was able to cause a significant depolarization, when ganglia were pretreated with L-NAME and 7NI.

To compare the effects of NOC-7 determined *in vitro* with those occurring *in situ*, we next performed experiments on B19 neurons located in the buccal ganglion. Somata of B19 neurons were investigated under whole-cell patch clamp conditions (see Materials and Methods). Interestingly, 100 µM NOC7 did not result in a depolarization of the membrane potential of B19 neurons within the ganglion (−0.3±0.2 mV, n = 5) [Fig. 8(B)]. Given the presence of NOS-containing and NO-producing neurons in the *Helisoma* buccal ganglion [12], we considered that B19 neurons *in situ* might already be exposed to significant concentrations of intrinsic NO, and that further addition of NOC-7 might therefore be ineffective. To test this hypothesis, we treated ganglia with NOS inhibitors with the rationale that once the intrinsic concentration of NO was reduced, addition of NOC-7 might have the same depolarizing effect on B19 neurons in ganglia as seen in cultured neurons above. To this end, buccal ganglia were incubated for 1 to 2 hours in a solution containing two NOS inhibitors, 1 mM L-NAME and 100 µM 7NI, known to inhibit NOS by different mechanisms [34]. Subsequent stimulation with the NO donor, NOC7 (100 µM), indeed significantly depolarized the membrane potential of B19 neurons (NOC7 after L-NAME&7NI: 3.3±0.5 mV, n = 4, P<0.05, compared to NOC7; Mann-Whitney U-test) [Fig. 8(A and B)]. Moreover, this level of depolarization was not significantly different from the depolarization seen in cultured B19 neurons (initial effect of NOC7 *in vitro*: +3.8±0.5 mV, n = 8, P = 0.80, compared to NOC7 after L-NAME&7NI in ganglia; Mann-Whitney U-test). NOC7 also increased the number of excitatory and inhibitory postsynaptic potentials received in B19 neurons, suggesting that NO had indirect effects on B19 neurons as well. Taken together, NO can depolarize B19 neurons in their ganglionic environment. Moreover, the concentration of NO produced within the ganglion might have already depolarized B19 neurons to a degree that prevented any additional effect of NOC7 in our experiments.

## Discussion

**Figure 9:**
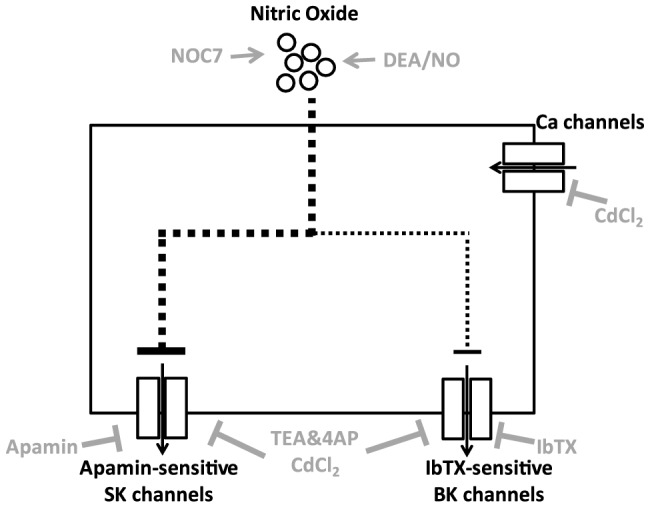
Proposed model of ion channel targets through which NO results in a prolonged depolarization. Elevation of NO by NO donors, such as NOC7 or DEA/NO, inhibits two types of Ca^2+^-activated K^+^ channels in *Helisoma* B19 neurons. Apamin-sensitive SK channels contribute to part of the initial effect of NO and are fully responsible for its long-lasting effect on membrane depolarization, whereas IbTX-sensitive BK channels only partially contribute to the initial depolarization. Voltage-gated Ca^2+^ channels do not participate in the depolarizing effect of extrinsically applied NO. The mechanism(s) by which NO inhibits these ion channels is presently unknown (indicated by dotted lines). Inhibitors used are indicated in gray.

The goal of current study was to understand the role of NO in modulating neuronal activity in B19 neurons from *Helisoma trivolvis*. We achieved this aim by investigating membrane channel targets that mediate the effects of NO at the electrophysiological level. The proposed model by which NO is thought to affect electrical activity in B19 neurons is schematically shown in Fig. 9. According to the model, NO depolarizes the membrane potential by inhibiting two types of Ca^2+^-activated K^+^ channels: apamin-sensitive K^+^ channels and IbTX-sensitive K^+^ channels, with the main effect of NO being contributed by the inhibition of apamin-sensitive K^+^ channels. NO application on the other hand had no significant effect on VGCCs.

### Effects of NO on membrane potential and cell excitability

Elevation of the NO concentration by treatment with the NO-donors NOC7 and DEA/NO led to a long-lasting depolarization of the membrane potential in B19 neurons. We divided this response into an initial phasic depolarization, followed by a tonic plateau response. The majority of B19 neurons were electrically silent before the stimulation with NO, and in most of these neurons, the NO-induced depolarization elicited transient or sustained spiking activity. Such a transition from a silent to a firing state constitutes a profound change in the physiological state of a neuron, regardless of whether a neuron is undergoing neurite outgrowth during development or regeneration, or serving as a member of a neuronal circuit in the mature nervous system. For example, neuronal spiking will increase the intracellular Ca^2+^ concentration ([Ca^2+^]_i_), which has been shown to have a wide range of effects in both developing and mature nervous systems [35]–[38]. Increases in [Ca^2+^]_i_ in growth cones from several neuron types have been shown to result in a decrease in neurite outgrowth [39], filopodial elongation [36], and growth cone turning [40]. In the intact nervous system, an increase in intrinsic spiking activity would result in altered postsynaptic excitation, and, depending on the degree of depolarization resulting from NO, it could lead to an increase or decrease in neuronal excitability [8], [41]. Even neurons that were originally silent, and in response to NO treatment became depolarized without reaching the spike threshold, would likely exhibit altered responses to presynaptic inputs.

In B5 neurons, we previously showed that NO had a biphasic effect, causing transient excitation, followed by silencing at a depolarized membrane potential [14]. In this case, NO caused an initial increase in firing frequency followed by a sustained depolarization, similar to that seen in B19 neurons. The difference between B5 and B19 neurons was that B5 neurons did not show a sustained increase in excitability in response to NO, whereas B19 neurons did show such an increase in excitability [14]. Therefore, the release of NO *in vivo* is expected to have complex effects on target neurons that may differ between cell types, depending on the mode of NO's action on individual neurons.

### Ion channels affected by NO

After ruling out the possibility that extrinsic NO might have opened VGCCs to cause depolarization, we found that the effect of NO on membrane potential was completely eliminated when K^+^ channels were inhibited with a cocktail of TEA and 4AP, supporting the hypothesis that K^+^ channels were primary targets of NO signaling. We next investigated any involvement of Ca^2+^-activated K^+^ channels by using CdCl_2_ to block VGCCs, with the rationale that Ca^2+^-activated K^+^ channels would be largely inhibited without Ca^2+^ influx [42]. Interestingly, we found that VGCCs, at resting conditions, contributed to the membrane potential, perhaps by activating Ca^2+^-activated K^+^ channels that help maintain a hyperpolarizing drive on the membrane potential. The membrane potential depolarized after the blockage of Ca^2+^ influx, suggesting the closure of Ca^2+^-activated K^+^ channels. The finding that NOC7, in the presence of CdCl_2_, was unable to elicit additional depolarization during the later phase indicated that Ca^2+^ influx and NOC7 signaling might be converging on a common target, such as Ca^2+^-activated K^+^ channels. In fact, NO signaling has been shown to inhibit Ca^2+^-activated K^+^ channels in various cells including *Helisoma* B5 neurons [14], [43].

Further pharmacological investigation of specific K^+^ channel subtypes suggested Ca^2+^-activated K^+^ channels, SK channels and BK channels, as the main ion channel targets of NO. This finding is consistent with what we reported in *Helisoma* B5 neurons [14], where NO regulates the electrical activity of tonically firing neurons through inhibition of SK channels and BK channels. Here, we further dissected the contributions of different channel inhibitors on the NO-induced membrane depolarization. The inhibition of SK channels with apamin resulted in an instant depolarization of the membrane potential, and this effect was sustained throughout the recording. The apamin-induced plateau depolarization was similar to that seen after NO treatment, and subsequent application of NOC7 did not show any additional effect on the plateau phase, suggesting that the plateau depolarization was most likely mediated by the closure of SK channels. However, NO still had a small depolarizing effect on membrane potential during the initial phase in the presence of apamin, although the level of depolarization was significantly smaller than that seen with NOC7 on its own. Interestingly, the initial effect of NO was also reduced when BK channels were blocked by IbTX. Taken together, these two findings suggested that the initial NO-induced depolarization could be explained by a combined effect of inhibition of both SK and BK channels by NO. Modulatory effects of NO on Ca^2+^-activated K^+^ channels were also reported in other cell types, including mammalian vascular smooth muscle [44], avian ciliary ganglia neurons [43], and other snail neurons [45], suggesting a conserved signaling role for NO on Ca^2+^-activated K^+^ channels.

Although the main targets of NO were most likely Ca^2+^-activated K^+^ channels, NO might also inhibit other K^+^ channels. In fact, a residual small depolarization by NO was still seen in the initial phase after inhibition of Ca^2+^ channels with CdCl_2_, which is thought to remove all contributions of Ca^2+^-activated K^+^ channels. NO has been shown to regulate various K^+^ channels [46]. For example, the delayed rectifier channel, Kv3, which regulates synaptic strength and intrinsic excitability, is inhibited by NO via volume transmission in the auditory brainstem and the hippocampus [8], [47]. Considering the important roles of K^+^ channels in determining action potential waveform [48], the modulatory effects of NO on K^+^ channels might not only have a strong impact on membrane potential but also tune the spike timing of these neurons.

### NO and gastropod feeding

NO is free to pass the plasma membrane and capable of acting on cellular targets in the vicinity of NO-releasing neurons, making it a good candidate for the modulation of neuronal circuits [6], [14]. We showed here that B19 neurons in the ganglion did not respond to stimulation with the NO donor NOC7. After incubation of ganglia with NOS inhibitors to eliminate endogenous NO release, however, treatment with NOC7 was able to depolarize the membrane potential of B19 neurons in ganglia to a similar degree as in cultured B19 neurons. This result has several interesting implications and will serve as a starting point for future experiments on the regulatory role of NO on the neuronal circuitry in the buccal ganglion and behaviors associated with it. First, the NO concentration present in buccal ganglia is apparently sufficiently high to depolarize the membrane potential of B19 neurons to a degree that additional treatment with NOC7 does not result in further depolarization. These results emphasize the need for comparative studies in culture and *in situ*. Such dual approach can be highly complementary and informative, as seen in the case of B19 neurons, where a study in the ganglion might have concluded incorrectly that B19 neurons do not respond to NO, where a study *in vitro* would have come to the opposite conclusion. The comparative approach suggests that B19 neurons actually react the same way *in situ* and *in vitro*, when adjusting for differences in the environment, such as the removal of tonic NO production in the ganglion. A study of physically isolated neurons in cell culture, therefore, provides a valuable approach to investigate aspects of a neuron's physiology in response to external stimulation without the complication from inputs from other cells. An early study of a nitrergic synapse between two motoneurons in *Lymnaea* demonstrated that nitrergic responses in neurons were maintained in the isolated neuronal culture condition [49]. In the case of B19 neurons, the effect of NOC7 on SK and BK channels actually can be best studied in cultured neurons, because the production of NO in the ganglion would have precluded such a study *in situ*. Thus, our findings make a strong argument that studies *in situ* and in culture can be highly complementary and may be necessary to fully characterize the electrical properties of individual neurons.

Inside the buccal ganglion, motor neuron B19 is active during the hyper-retraction phase (S3) of the feeding motor pattern in *Helisoma* and activates several muscle groups in the radula [26], [27]. Physiological release of NO, either through nitrergic neurons projecting into the buccal ganglion, or from neurons located within the ganglion [12], [50], are expected to depolarize B19 neurons, resulting in an increase in their firing frequency and membrane excitability. Moreover, NO signaling would likely alter the response of B19 neurons to presynaptic inputs, and we show an increase in inhibitory inputs into B19 neurons in response to NO. NO has been shown to enhance the synaptic strength of serotonergic neurotransmission between the cerebral giant cell and the buccal neuron B4 in *Lymnaea* [51]. This effect could potentially be explained by the NO-induced increase in excitability, which would strengthen the influence of the cerebral giant cell on the feeding motor patterns.

How NO signaling would affect overall snail feeding is presently unclear. NO has been described as a regulator for the feeding motor patterns in *Lymnaea* [24]. An early study showed that the treatment with a NO donor activates feeding movements of the buccal mass [23], whereas a more recent study reported that NO release *in situ* functions to suppress rhythmic activity in buccal motor neurons, resulting in a reduced feeding rate [22]. These seemingly opposing effects of NO on snail feeding warrant future investigations on the effects of NO on multiple levels, including studies on isolated neurons, the neuronal circuitry generating the feeding motor program, and animal behavior.
